# lncRNA RPSAP52 induced the development of tongue squamous cell carcinomas via miR‐423‐5p/MYBL2

**DOI:** 10.1111/jcmm.16442

**Published:** 2021-03-30

**Authors:** Xiaozhen Wu, Zuode Gong, Long Ma, Qibao Wang

**Affiliations:** ^1^ Department of stomatology Aerospace Center Hospital Beijing China; ^2^ Department of Endodontics Jinan Stomatological Hospital Jinan China

**Keywords:** miR‐423‐5p, MYBL2, RPSAP52, tongue squamous cell carcinomas

## Abstract

Growing lncRNAs have been noted to involve in the initiation and development of several tumours including tongue squamous cell carcinomas (TSCCs). However, the biological role and mechanism of lncRNA RPSAP52 were not well‐explained. We indicated that RPSAP52 was higher in TSCC samples compared with that in control samples. The higher expression of RPSAP52 was positively correlated with higher T stage and TNM stage. Ectopic expression of RPSAP52 induced TSCC cell growth and cycle and induced cytokine secretion including IFN‐γ, IL‐1β and IL‐6, IL‐8, IL‐10 and TGF‐β. We found that the overexpression of RPSAP52 suppressed miR‐423‐5p expression in SCC‐4 cell. miR‐423‐5p was lower in TSCC samples compared with that in control samples, and miR‐423‐5p level was negatively correlated with higher T stage and TNM stage. Pearson's correlation indicated that miR‐423‐5p was negatively associated with that of RPSAP52 in TSCC tissues. Furthermore, MYBL2 was one direct gene of miR‐423‐5p and elevated expression of miR‐423‐5p suppressed MYBL2 expression and ectopic expression of RPSAP52 increased MYBL2 expression in SCC‐4 cell. Finally, we illustrated that RPSAP52 overexpression promoted TSCC cell growth and cycle and induced cytokine secretion including IFN‐γ, IL‐1β and IL‐6, IL‐8, IL‐10 and TGF‐β via modulating MYBL2. These data provided new insight into RPSAP52, which may be one potential treatment target for TSCC.

## 1 INTRODUCTION

Oral squamous cell carcinomas (OSCCs) are understudied and undertreated diseases.^1^, ^2^, ^3^, ^4^, ^5^ SCC of tongue (TSCC) is the most frequent carcinoma of OSCC, with aspects of rapid metastatic spread and local invasion.^6^, ^7^, ^8^ The incidence of these diseases is really grown in middle and young age populations.^9^, ^10^, ^11^ Although treatment attempts such as surgery, radiotherapy and chemotherapy have been tried, the prognosis of TSCC remains unsatisfactory.^12^, ^13^, ^14^, ^15^ It suggested that it is one of the major health issues and it needs the understanding of molecular mechanisms of progression or carcinogenesis of this disease. It will be helpful for improving prevention, therapy and diagnosis of TSCC.

lncRNAs were loosely regarded as new RNA molecules, which were more than 200 nts and lack protein‐coding ability.^16^, ^17^, ^18^, ^19^, ^20^ Deregulated expression of lncRNAs is associated with several diseases including respiratory, neurodegenerative and cardiovascular diseases, and scoliosis.^21^, ^22^, ^23^, ^24^ Numerous references illuminated that lncRNAs participate in critical cell processes including cell metabolism, invasion, apoptosis, proliferation and differentiation.^25^, ^26^, ^27^, ^28^ Increasing studies also noted that lncRNAs are involved in the development and progression of TSCC.^29^, ^30^ Recently, a new lncRNA RPSAP52 has been identified and revealed to participate in several tumours such as glioblastoma, pituitary tumours and pancreatic cancer.^31^, ^32^, ^33^, ^34^ However, the biological role and mechanism of lncRNA RPSAP52 were not well‐explained.

## 2 MATERIALS AND METHODS

### 2.1 Patient's samples and cell culture and transfection

TSCC and their morphological control specimens were collected from 30 TSCC cases undergoing surgery at Jinan Stomatological Hospital. Specimens were immediately frozen in liquid nitrogen until RNA was used. All protocols were agreed with the Ethics Committee of Jinan Stomatological Hospital, and all patients signed the informed consent. TSCC lines (UM1, SCC‐4, SCC‐1 and Cal27) and NHOK were obtained from ATCC. Cells were cultured in the DMEM supplemented with FBS, penicillin and streptomycin. pcDNA‐RPSAP52 and MYBL2 siRNA vector, miRNA mimic and control plasmids were synthesized by GenePharma and then transfected into cells using Lipofectamine 3000 (Invitrogen).

### 2.2 Quantitative PCR

Total RNA from TSCC tissues and cells was extracted using TRIzol Kit (Invitrogen) following the instructions. This primer was designed as follows: RPSAP52: F, 5‐′GAG CAA ACA CAT CGG AGACA‐3′, and R, 5‐′AAT TGG ATT CCC ACTG CAAG‐3′; GAPDH: F, 5‐′GACCTG ACC TGC CGT CTA G‐3′, and R, 5‐′AGG AGT GGG TGT CGC TGT‐3′; U6: F, 5′‐CTCGCT TCGGCA GCACA‐3′, and R, 5′‐AACGCT TCA CGAATT TGCGT‐3′; MYBL2: F, 5‐′GAGGG ATAGC AAGTG CAAGGT‐3′, and R, 5‐′TTCCA GTCCT GCTGTC CAAA‐3′; and miR‐423‐5p: F, 5‐′TGAGG GGCAG AGCGA GACTTT‐3′, and R, 5‐′GTGCA GGGTCC GAGGTGG GCAGAG CGAGACTTT‐3′. qRT‐PCR analysis was conducted with SYBR Premix I (Takara) on the ABI 7900HT qRT‐PCR System (Bio‐Rad). The relative value of the miRNA, lncRNA and mRNA expression was analysed using 2^−△△Ct^ method. U6 and GAPDH acted as control genes for miRNA and lncRNA and mRNA, respectively.

### 2.3 Cell proliferation and ELISA

Different groups of TSCC cells were plated in 96‐well plates. Cell growth was determined by CCK‐8 method, and 10 μL CCK‐8 reagent was added into each well until visual colour was changed. The absorbance was determined by the microplate reader at 450 nM. Cytokine level was detected using ELISA reagents (R&D Systems). The absorbance at 450 nm was recorded by the microplate reader.

### 2.4 Luciferase reporter gene assay

Cells were cultured in 96‐well dish. The mutant sequence of MYBL2 (pmirGLO‐MYBL2‐Mut) and its wild sequence (pmirGLO‐MYBL2‐wt) were cloned into luciferase reporter and then named as pmirGLO‐MYBL2‐Mut and pmirGLO‐MYBL2‐WT, respectively. Cells were transfected with mimic and scramble and pmirGLO‐MYBL2‐Mut and pmirGLO‐MYBL2‐WT by Lipofectamine 3000 (Invitrogen). Forty‐eight hours later, cells were harvested and luciferase value was detected by dual‐luciferase analysis (Promega) following the protocol.

### 2.5 Statistical analysis

The statistical assay was conducted by SPSS 21.0 software, and results were presented as mean ± SD. The difference between 2 groups was evaluated by *t* test, and *P* value < .05 was considered to be significant. The correlation between miR‐423‐5p and RPSAP52 was analysed by Pearson's correlation.

## 3 RESULTS

### 3.1 RPSAP52 was overexpressed in TSCC samples

**FIGURE 1:**
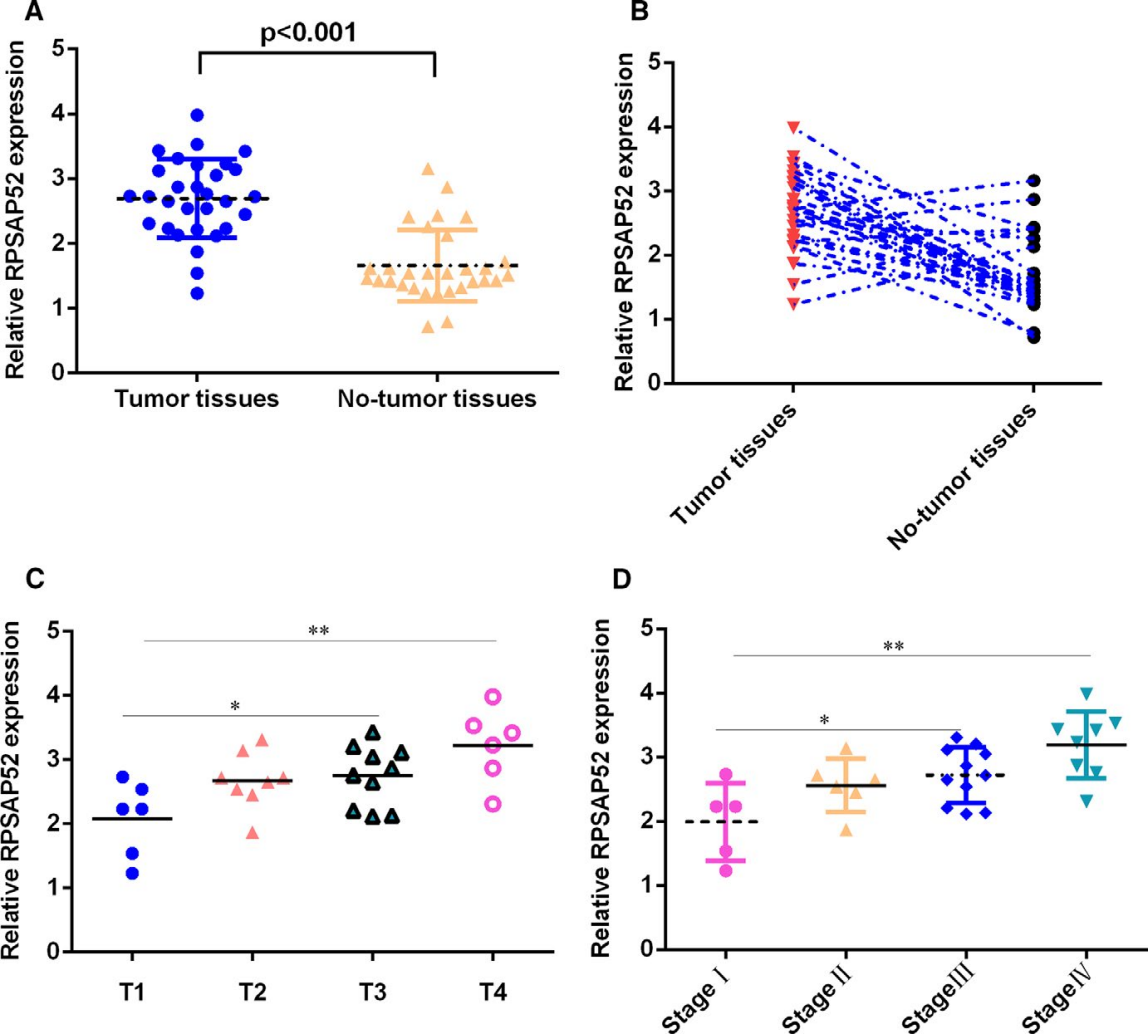
RPSAP52 was overexpressed in TSCC samples. (A) RT‐qPCR data suggested that RPSAP52 was higher in TSCC samples compared with that in control samples. (B) RPSAP52 was overexpressed in 25 TSCC cases (25/30, 83.3%) compared with control no‐tumour samples. (C) The higher expression of RPSAP52 was positively correlated with higher T stage. (D) The higher expression of RPSAP52 was positively correlated with TNM stage. GAPDH was used as internal control. *P < .05 and **P < .01

RT‐qPCR data suggested that RPSAP52 was higher in TSCC samples compared with that in control samples (Figure 1A). RPSAP52 was overexpressed in 25 TSCC cases (25/30, 83.3%) compared with control no‐tumour samples (Figure 1B). The higher expression of RPSAP52 was positively correlated with higher T stage (Figure 1C) and TNM stage (Figure 1D).

### 3.2 miR‐423‐5p was down‐regulated in TSCC specimens

**FIGURE 2:**
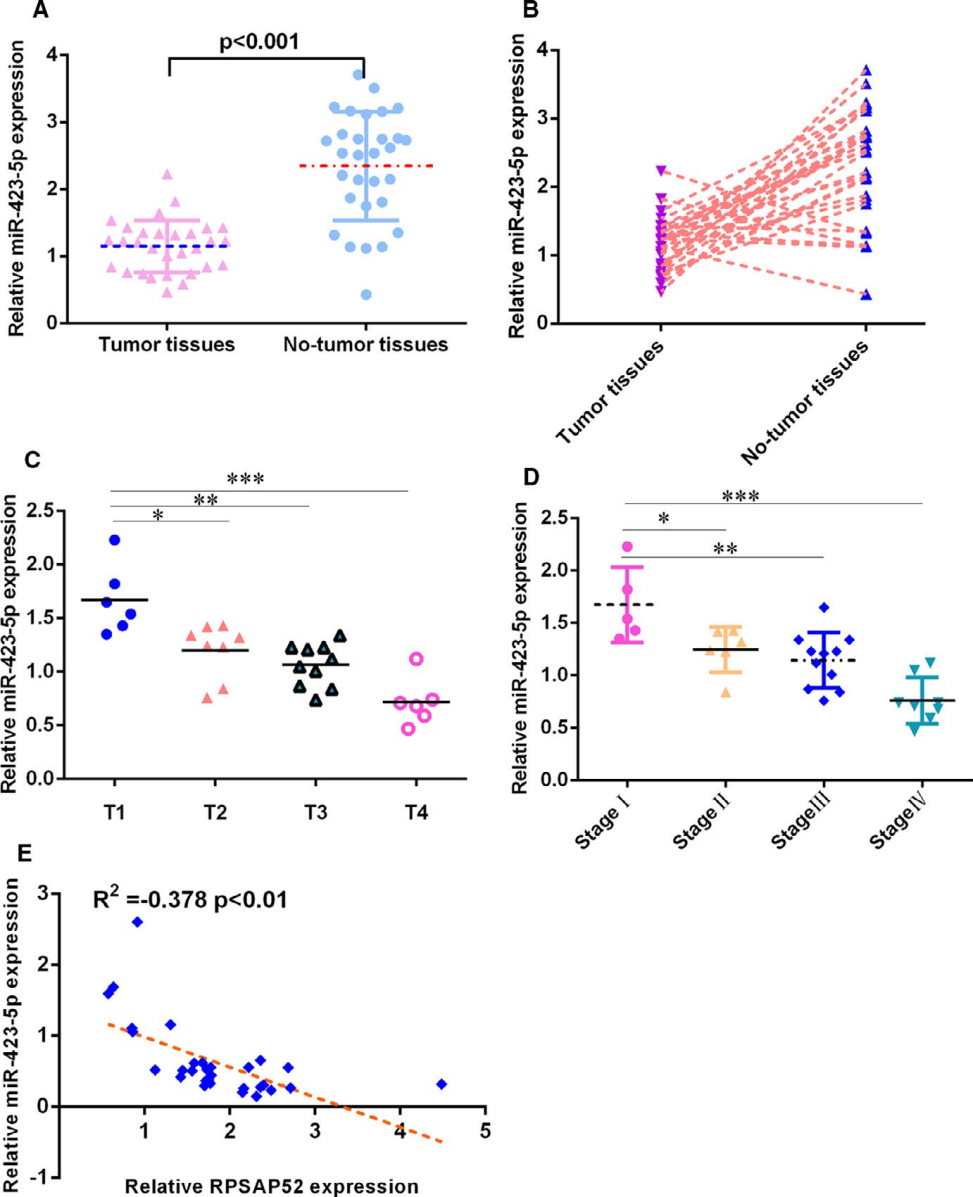
miR‐423‐5p was down‐regulated in TSCC specimens. (A) miR‐423‐5p was lower in TSCC samples compared with that in control samples. (B) miR‐423‐5p was down‐regulated in 24 TSCC cases (24/30, 80%) compared with control no‐tumour samples. (C) The miR‐423‐5p level was negatively correlated with higher T stage. (D) The miR‐423‐5p level was negatively correlated with TNM stage. (E) Pearson's correlation indicated that miR‐423‐5p was negatively associated with that of RPSAP52 in TSCC tissues. *P < .05, **P < .01 and ***P < .001

RT‐qPCR assay showed that miR‐423‐5p was lower in TSCC samples compared with that in control samples (Figure 2A). miR‐423‐5p was down‐regulated in 24 TSCC cases (24/30, 80%) compared with control no‐tumour samples (Figure 2B). The miR‐423‐5p level was negatively correlated with higher T stage (Figure 2C) and TNM stage (Figure 2D). Pearson's correlation indicated that miR‐423‐5p was negatively associated with that of RPSAP52 in TSCC tissues (Figure 2E).

### 3.3 Ectopic expression of RPSAP52 induced TSCC cell growth

**FIGURE 3:**
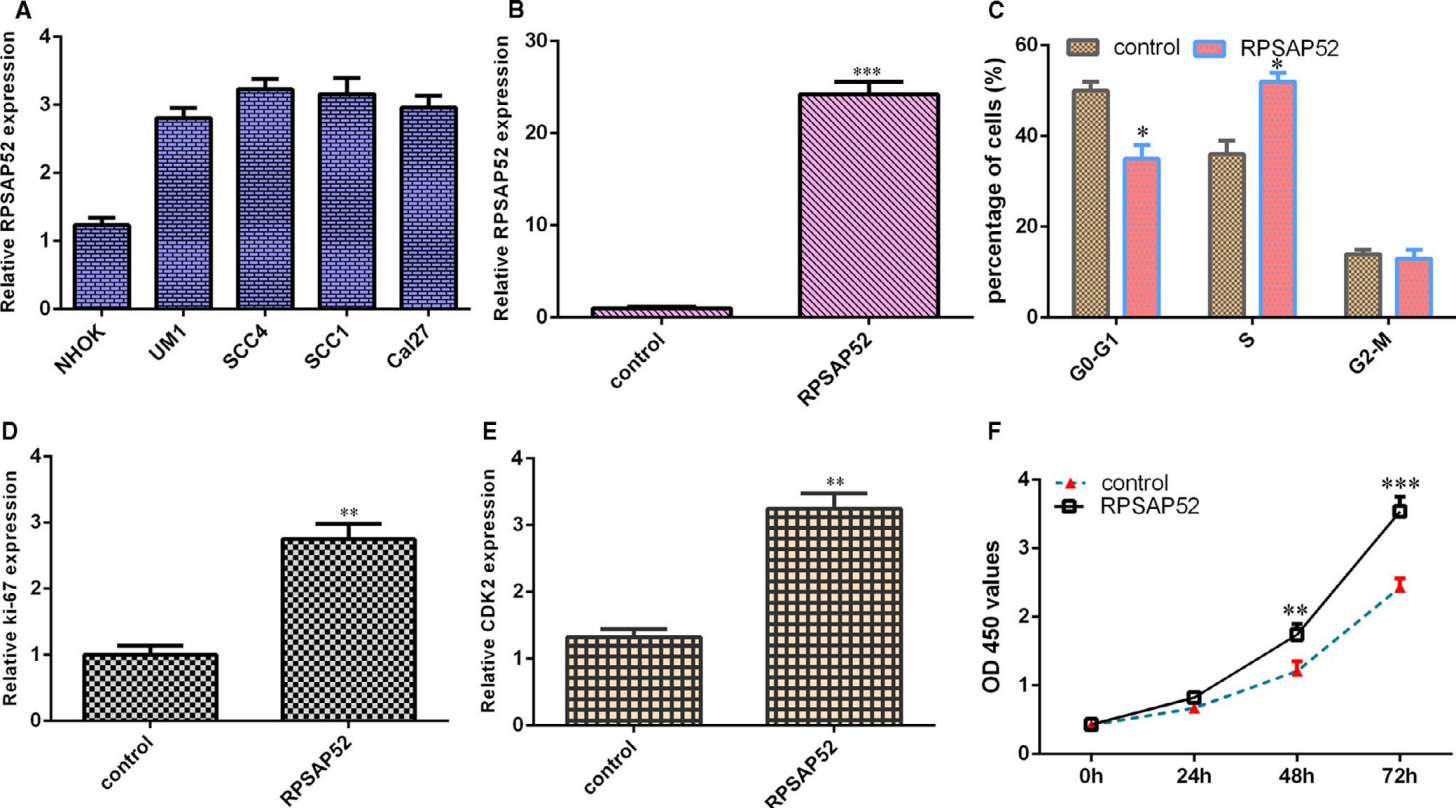
Ectopic expression of RPSAP52 induced TSCC cell growth. (A) RPSAP52 level was overexpressed in four TSCC lines (UM1, SCC‐4, SCC‐1 and Cal‐27) compared with NHOK cell. (B) The expression of RPSAP52 was detected by qRT‐PCR. (C) Elevated expression of RPSAP52 increased cells at S stage and decreased cells at G0‐G1 stage. (D) Overexpression of RPSAP52 induced ki‐67 expression in SCC‐4 cell. (E) The expression of CDK2 was measured by qRT‐PCR analysis. (F) Ectopic RPSAP52 expression increased cell growth in SCC‐4 cell. *P < .05, **P < .01 and ***P < .001

RPSAP52 level was overexpressed in four TSCC lines (UM1, SCC‐4, SCC‐1 and Cal‐27) compared with NHOK cell (Figure 3A). RPSAP52 level was strikingly up‐regulated in SCC‐4 after transfected with pcDNA‐RPSAP52 (Figure 3B). Elevated expression of RPSAP52 increased cells at S stage and decreased cells at G0‐G1 stage (Figure 3C). Overexpression of RPSAP52 induced ki‐67 (Figure 3D) and CDK2 (Figure 3E) expression in SCC‐4 cell. Ectopic RPSAP52 expression increased cell growth in SCC‐4 cell (Figure 3F).

### 3.4 Elevated expression of RPSAP52 induced cytokine secretion in TSCC cell

**FIGURE 4:**
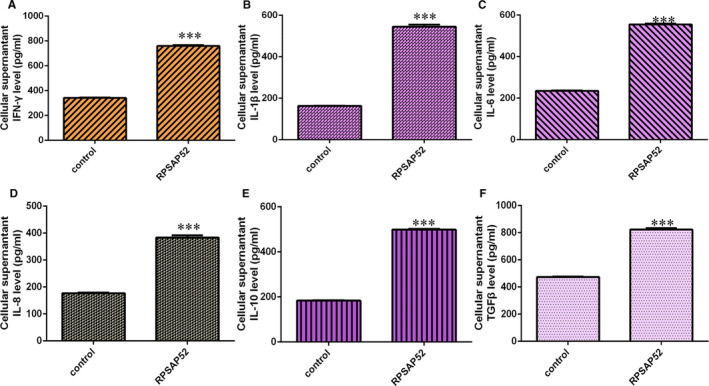
Elevated expression of RPSAP52 induced cytokine secretion in TSCC cell. (A) The expression of IFN‐γ was determined by ELISA. (B) The expression of IL‐1β was detected using ELISA. (C) The expression of IL‐6 was detected using ELISA. (D) The expression of IL‐8 was determined by ELISA. (E) The expression of IL‐10 was detected using ELISA. (F) The expression of TGF‐β was detected using ELISA. ***P < .001

The cytokine concentrations of IFN‐γ (Figure 4A), IL‐1β (Figure 4B) and IL‐6 (Figure 4C) were up‐regulated in SCC‐4 cell after treated with pcDNA‐RPSAP52. Ectopic expression of RPSAP52 promoted cytokine concentration excretion including IL‐8 (Figure 4D), IL‐10 (Figure 4E) and TGF‐β (Figure 4F) expression.

### 3.5 MYBL2 was one direct gene of miR‐423‐5p

**FIGURE 5:**
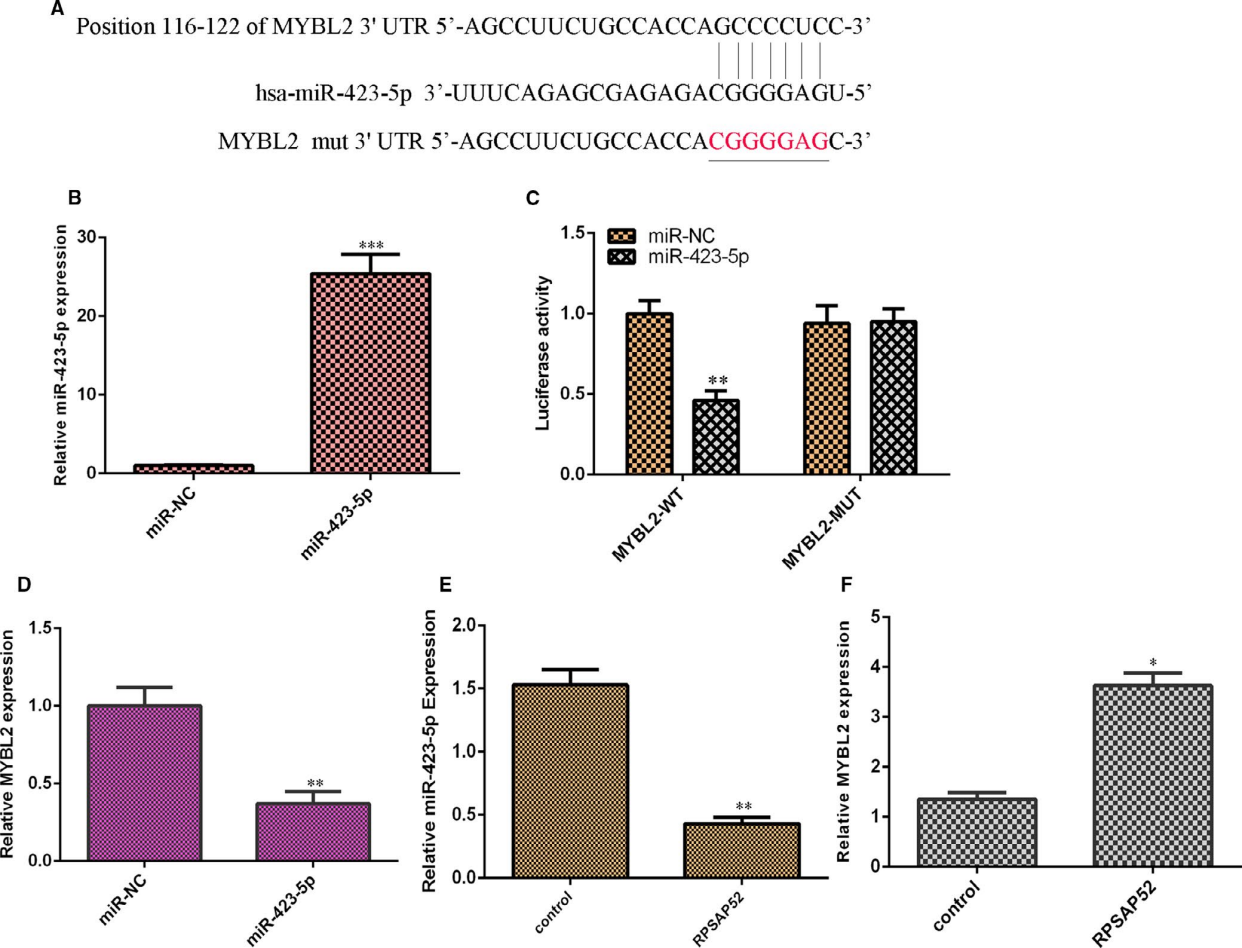
MYBL2 was one direct gene of miR‐423‐5p. (A) Bioinformatic TargetScan assay noted that MYBL2 contained target sites of miR‐423‐5p. (B) The level of miR‐423‐5p was determined by qRT‐PCR. (C) Luciferase reporter assay suggested the ectopic expression of miR‐423‐5p attenuated luciferase value of MYBL2‐WT reporter vector, but not that of MYBL2‐mut reporter vector in SCC‐4 cell. (D) Elevated expression of miR‐423‐5p suppressed MYBL2 expression in SCC‐4 cell. (E) Overexpression of RPSAP52 suppressed miR‐423‐5p expression in SCC‐4 cell. (F) Ectopic expression of RPSAP52 increased MYBL2 expression in SCC‐4 cell. **P < .01

One bioinformatic TargetScan assay (http://www.targetscan.org/vert_72/) noted that MYBL2 contained target sites of miR‐423‐5p as indicated in Figure 5A. The level of miR‐423‐5p was strikingly overexpressed in SCC‐4 cell after transfection with miR‐423‐5p mimic (Figure 5B). Luciferase reporter assay suggested that ectopic expression of miR‐423‐5p attenuated luciferase value of MYBL2‐WT reporter vector, but not that of MYBL2‐mut reporter vector in SCC‐4 cell (Figure 5C). Elevated expression of miR‐423‐5p suppressed MYBL2 expression in SCC‐4 cell (Figure 5D). Overexpression of RPSAP52 suppressed miR‐423‐5p expression in SCC‐4 cell (Figure 5E). Ectopic expression of RPSAP52 increased MYBL2 expression in SCC‐4 cell (Figure 5F).

### 3.6 Ectopic expression of RPSAP52 increased cell growth via modulating MYBL2 in TSCC cell

**FIGURE 6:**
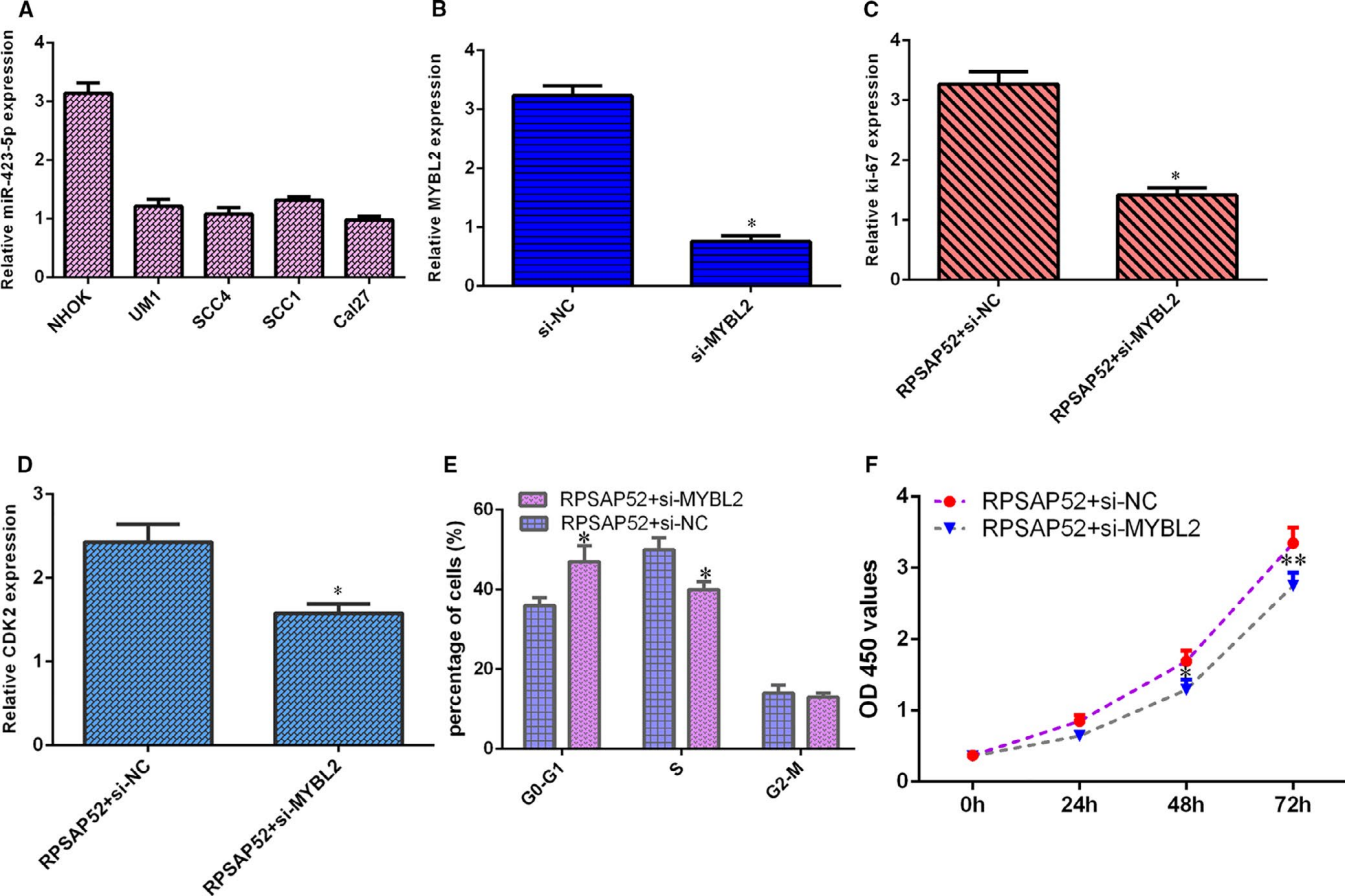
Ectopic expression of RPSAP52 increased cell growth via modulating MYBL2 in TSCC cell. (A) miR‐423‐5p level was down‐regulated in four TSCC lines (UM1, SCC‐4, SCC‐1 and Cal‐27) compared with NHOK cell. (B) The expression of MYBL2 was determined by qRT‐PCR analysis. (C) The expression of ki‐67 was studied by qRT‐PCR analysis. (D) The expression of CDK2 was studied using qRT‐PCR analysis. (E) Down‐regulation of MYBL2 suppressed cells at S stage and increased cells at G0‐G1 stage in RPSAP52‐overexpressing SCC‐4 cell. (F) Knockdown expression of MYBL2 inhibited cell proliferation in RPSAP52‐overexpressing SCC‐4 cell. *P < .05 and **P < .01

miR‐423‐5p level was down‐regulated in four TSCC lines (UM1, SCC‐4, SCC‐1 and Cal‐27) compared with NHOK cell (Figure 6A). The expression of MYBL2 was down‐regulated in SCC‐4 cell after transfected with MYBL2 siRNA (Figure 6B). Knockdown expression of MYBL2 suppressed ki‐67 (Figure 6C) and CDK2 (Figure 6D) expression in RPSAP52‐overexpressing SCC‐4 cell. Down‐regulation of MYBL2 suppressed cells at S stage and increased cells at G0‐G1 stage in RPSAP52‐overexpressing SCC‐4 cell (Figure 6E). Knockdown expression of MYBL2 inhibited cell proliferation in RPSAP52‐overexpressing SCC‐4 cell (Figure 6F).

### 3.7 RPSAP52 induced cytokine secretion through regulating MYBL2 in TSCC cell

**FIGURE 7:**
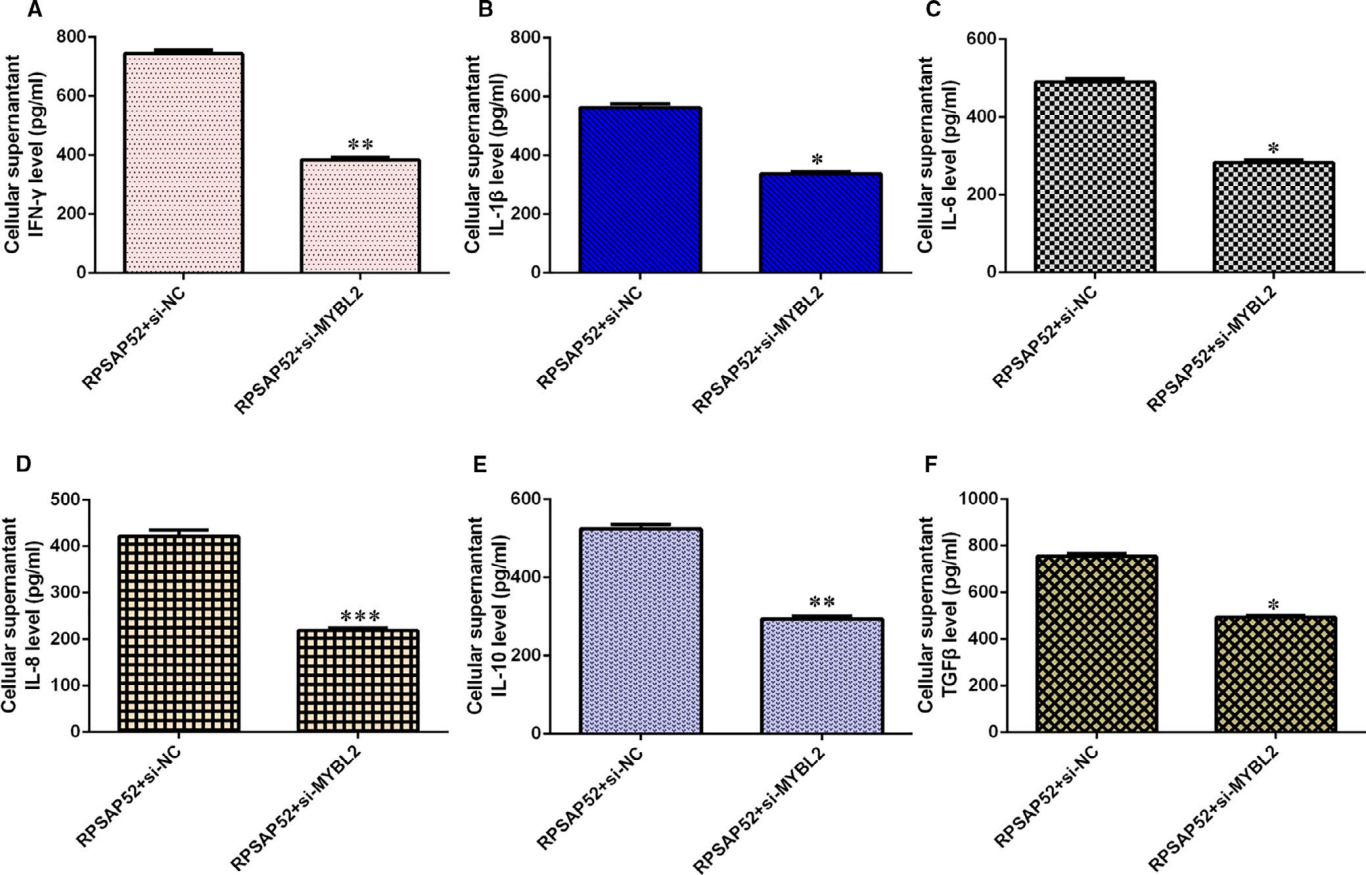
RPSAP52 induced cytokine secretion through regulating MYBL2 in TSCC cell. (A) The expression of IFN‐γ was determined by ELISA. (B) The expression of IL‐1β was detected using ELISA. (C) The expression of IL‐6 was detected using ELISA. (D) The expression of IL‐8 was determined by ELISA. (E) The expression of IL‐10 was detected using ELISA. (F) The expression of TGF‐β was detected using ELISA. *P < .05, **P < .01 and ***P < .001

The cytokine concentrations of IFN‐γ (Figure 7A), IL‐1β (Figure 7B) and IL‐6 (Figure 7C) were decreased in SCC‐4 cell after treatment with MYBL2 siRNA in RPSAP52‐overexpressing SCC‐4 cell. Knockdown expression of MYBL2 suppressed cytokine concentration excretion including IL‐8 (Figure 7D), IL‐10 (Figure 7E) and TGF‐β (Figure 7F) expression.

## 4 DISCUSSION

Growing lncRNAs have been noted to involve in the initiation and development of several tumours including TSCC. For instance, Zhang et al^35^ indicated that TUG1 level was overexpressed in TSCC cells and tissues of cisplatin resistance. Knockdown of TUG1 suppressed cisplatin resistance to CAL27/CDDP and SCC25/CDDP cells via modulating CXCR4 and miR‐133b. Lin et al^36^ noted that PRNCR1 played as one oncogenic lncRNA gene in the development of TSCC via regulating HOXB5/ miR‑944. Yan et al^37^ suggested that knockdown of PCAT‐1 suppressed metastasis of proliferation of TSCC cell through up‐regulating P21. Liu and workmates found that knockdown of SNHG17 suppressed TSCC cell migration, invasion and proliferation through regulating miR‐876/SP1.^38^ Recently, a new lncRNA RPSAP52 has been identified and revealed to participate in several tumours such as glioblastoma, pituitary tumours and pancreatic cancer.^31^, ^32^, ^33^, ^34^ Wang et al^33^ showed that RPSAP52 predicted postoperative survival and promoted cell stemness in glioblastoma through regulating TGF‐β1. D'Angelo et al^31^ found that RPSAP52 was up‐regulated in pituitary cancers and induced cell growth through sponging HMGA. However, the role of RPSAP52 remains unknown. In our study, we indicated that RPSAP52 was higher in TSCC samples compared with that in control samples. The higher expression of RPSAP52 was positively correlated with higher T stage and TNM stage. Ectopic expression of RPSAP52 induced TSCC cell growth and cycle and induced cytokine secretion including IFN‐γ, IL‐1β and IL‐6, IL‐8, IL‐10 and TGF‐β.

Emerging references illustrated that lncRNAs acted as ‘sponges’ of miRNAs to involve in several cellular processes. For example, Qiao et al^39^ showed that KCNQ1OT1 regulated cisplatin resistance of TSCC via TRIM14/miR‐124‐3p axis. Li et al^40^ illustrated that ADAMTS9‐AS2 enhanced TSCC migration, EMT and growth through EZH2/miR‐600 axis. Ma et al^41^ suggested that GIHCG induced TSCC progression via modulating miR‐429. Zuo et al^42^ proved that CASC15 induced TSCC development via sponging miR‐33a‐5p. Kou et al^43^ noted that H19 facilitated TSCC invasion and migration through regulating miR‐let‐7. Recently, Chen et al^34^ found that RPSAP52 inhibited hypoxia‐influenced epithelial cell apoptosis of renal proximal tubular via GSTM1/miR‐423‐5p axis. We also found that the overexpression of RPSAP52 suppressed miR‐423‐5p expression in SCC‐4 cell. miR‐423‐5p was lower in TSCC samples compared with that in control samples, and miR‐423‐5p level was negatively correlated with higher T stage and TNM stage. Pearson's correlation indicated that miR‐423‐5p was negatively associated with that of RPSAP52 in TSCC tissues. Furthermore, MYBL2 was one direct gene of miR‐423‐5p and elevated expression of miR‐423‐5p suppressed MYBL2 expression and ectopic expression of RPSAP52 increased MYBL2 expression in SCC‐4 cell. A previous study indicated that MYBL2 acted as one oncogene in tumour development.^44^, ^45^ Finally, we illustrated that RPSAP52 overexpression promoted TSCC cell growth and cycle and induced cytokine secretion including IFN‐γ, IL‐1β and IL‐6, IL‐8, IL‐10 and TGF‐β via modulating MYBL2.

In summary, we identified that RPSAP52 was up‐regulated in TSCC samples and positively correlated with higher T stage and TNM stage. Ectopic expression of RPSAP52 induced TSCC cell growth and cycle and induced cytokine secretion including IFN‐γ, IL‐1β and IL‐6, IL‐8, IL‐10 and TGF‐β through regulating miR‐423‐5p/ MYBL2 axis. These data provided new insight into RPSAP52, which may be one potential treatment target for TSCC.

## CONFLICT OF INTEREST

There is no conflict of interest.

## AUTHOR CONTRIBUTIONS

**Xiaozhen Wu:** Conceptualization (equal); Data curation (equal); Writing‐original draft (equal); Writing‐review & editing (equal). **Zuode Gong:** Conceptualization (equal); Investigation (equal); Resources (equal); Writing‐original draft (equal); Writing‐review & editing (equal). **Long Ma:** Conceptualization (equal); Data curation (equal); Investigation (equal); Writing‐original draft (equal). **Qibao Wang:** Conceptualization (equal); Data curation (equal); Funding acquisition (equal); Project administration (equal); Writing‐original draft (equal); Writing‐review & editing (equal).

## Data Availability

The data will be made available after being required upon request from the corresponding author.
